# Influence of Microbial Biofilms on the Preservation of Primary Soft Tissue in Fossil and Extant Archosaurs

**DOI:** 10.1371/journal.pone.0013334

**Published:** 2010-10-12

**Authors:** Joseph E. Peterson, Melissa E. Lenczewski, Reed P. Scherer

**Affiliations:** 1 Department of Geology, University of Wisconsin-Oshkosh, Oshkosh, Wisconsin, United States of America; 2 Department of Geology and Environmental Geosciences, Northern Illinois University, DeKalb, Illinois, United States of America; Paleontological Institute, Russian Federation

## Abstract

**Background:**

Mineralized and permineralized bone is the most common form of fossilization in the vertebrate record. Preservation of gross soft tissues is extremely rare, but recent studies have suggested that primary soft tissues and biomolecules are more commonly preserved within preserved bones than had been presumed. Some of these claims have been challenged, with presentation of evidence suggesting that some of the structures are microbial artifacts, not primary soft tissues. The identification of biomolecules in fossil vertebrate extracts from a specimen of *Brachylophosaurus canadensis* has shown the interpretation of preserved organic remains as microbial biofilm to be highly unlikely. These discussions also propose a variety of potential mechanisms that would permit the preservation of soft-tissues in vertebrate fossils over geologic time.

**Methodology/Principal Findings:**

This study experimentally examines the role of microbial biofilms in soft-tissue preservation in vertebrate fossils by quantitatively establishing the growth and morphology of biofilms on extant archosaur bone. These results are microscopically and morphologically compared with soft-tissue extracts from vertebrate fossils from the Hell Creek Formation of southeastern Montana (Latest Maastrichtian) in order to investigate the potential role of microbial biofilms on the preservation of fossil bone and bound organic matter in a variety of taphonomic settings. Based on these analyses, we highlight a mechanism whereby this bound organic matter may be preserved.

**Conclusions/Significance:**

Results of the study indicate that the crystallization of microbial biofilms on decomposing organic matter within vertebrate bone in early taphonomic stages may contribute to the preservation of primary soft tissues deeper in the bone structure.

## Introduction

Recent reports of the preservation of non-biomineralized proteins and tissues such as blood vessels in fossil bone has had a major impact on our understanding of fossil preservation processes [1], [2], [3]. Previous research results have claimed the presence of primary soft tissues, in the form of vessels, blood cells, bone cells by acid demineralization of a *Tyrannosaurus rex* femur [1], with subsequent reports of the preservation of protein sequences in *Tyrannosaurus rex* and *Mammut americanum* [2] and in the Campanian hadrosaurid *Brachylophosaurus canadesis* [3]. An alternative hypothesis for these findings has been presented, suggesting that pliable demineralized extracts from fossil bone more likely represents non-mineralized microbial biofilms rather than primary soft-tissues [4].

This study investigates the role of microbial biofilms on the preservation of primary soft-tissues. Presented here are the results of a controlled experiment in which modern biofilms were grown in extant archosaur bone under distinctly different environmental conditions. The aim of this experiment was to explore variation in microbial communities in decomposing bone, and the impact of microbial communities on soft tissue preservation. The main objectives of this study were to identify the characteristics of biofilms that develops as extant archosaur bone decays, and to identify the structures that are present in fossil dinosaur bone before and after demineralization.

Most biostratinomic process studies on vertebrate fossils have focused on mechanical transport of vertebrate remains, and have shown that variability of fossil dispersal is related to a variety of factors, notably hydrodynamic properties of the remains and preservation potential [5]–[9]. These factors directly affect the distribution of fossils in a stratum [5]. Similarly, the rate at which a submerged or subaereally exposed vertebrate carcass will fully decompose is greatly influenced by numerous physical and chemical factors including predation, ambient temperature and humidity, and a variety of other environmental conditions that influence rates of decay [10].

In most well-aerated soils, organic material biodegrades very rapidly. However, due to the high mineral content and relatively low permeability of bone, microbial decomposition of collagen within the bone is initially inhibited [10]. In order to biodegrade bone, microbes must be able to obtain energy from the mineral and collagen components of bone, which is achieved by demineralizing the bone or growing in an environment where demineralization naturally occurs, such as low pH soils [10]. Microorganisms penetrate the outermost surface of the bone itself through the natural permeability of the bone surface, especially via blood vessels and nerve lacunae, and microorganisms may form biofilms along the walls of these foraminae [10].

Biofilm is a general term applied to a variety of matrix-enclosed bacterial populations adherent to each other and/or to wet or moist surfaces or interfaces [11]. This includes microbial aggregates and floccules and also adherent populations within the pore spaces of porous media. In an aqueous environment, planktonic bacterial cells land on a surface and phenotypically react to the conditions of the medium; in high-nutrient conditions reverting to a sessile morphology and excreting an exopolysaccharide (EPS) which allows the bacteria to stick to its' substrate and other cells as it reproduces. Biofilms may consist of one or many species of microorganisms, all incorporated into the community. As the biofilm matures, it continues to reproduce. Biofilm growth is influenced by the available nutrients at the attachment site. As microorganisms metabolize their base, they change the environmental conditions by raising or lowering pH, which may cause the microorganisms to produce mineral precipitates such as calcium phosphate (hydroxyapatite) and calcium carbonate, depending on the composition of the substrate [12]. An example of this process occurs in Foley urinary catheters, where encrusting calcium-phosphate crystalline biofilms can develop [13]. In many cases this biofilm encrustation occurs to such severity as to cause complete blockage of flow through the catheters.

The identification of biomolecules with vertebrate signatures [2], [3] in the extracts from fossil vertebrates provides compelling evidence countering the argument that these structures are simply microbial biofilm [4]. Many of these reports have proposed a variety of potential preservation mechanisms [14]–[16]. The data presented here provides another potential mechanism for allowing enhanced soft-tissue preservation by exploring the role of microbial biofilms in sealing off and preserving primary soft tissues, a process we call “microbial masonry”.

## Materials and Methods

To investigate the impact of microorganisms on the preservation of soft-tissues in dinosaur bone, modern bone samples were obtained as substrates for biofilm development. To more accurately model dinosaur bone, Witmer's Extant Phylogenetic Bracket method of inference [17] was applied, leading to the choice of extant samples of archosauria as experimental media. These include both birds and crocodilians, in this case the bones of *Gallus gallus* (Domestic Chicken) and *Alligator mississipiensis* (American Alligator).

Bones chosen for the experiment consisted of long bones, such as femora and tibiae from both species. To specifically observe the growth of biofilm on bone and to expedite the process, bones were stripped of a majority of muscle and connective tissues, and placed in traps made of 250mL Nalgene bottles with holes drilled to allow water to move freely in and out of the bottle, but inhibit larger organisms from interacting with the specimens. Both a complete and bisected long bone was placed in each trap. This differentiation was intended to investigate differences in bone condition prior to microbial interaction. While freeze-thaw cycles may have an influence on bone degradation, sample chambers were frozen and stored at −70°C until deployment in order to minimalize rapid decomposition of the samples and minimize microbial cross-contamination.

**Figure 1:**
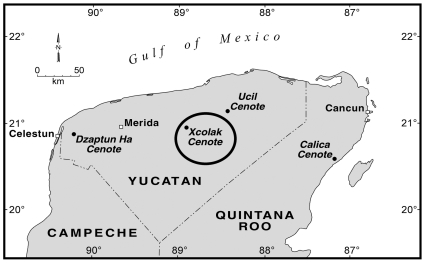
Location of Xcolak Cenote where samples were placed for biofilm growth.

**Figure 2:**
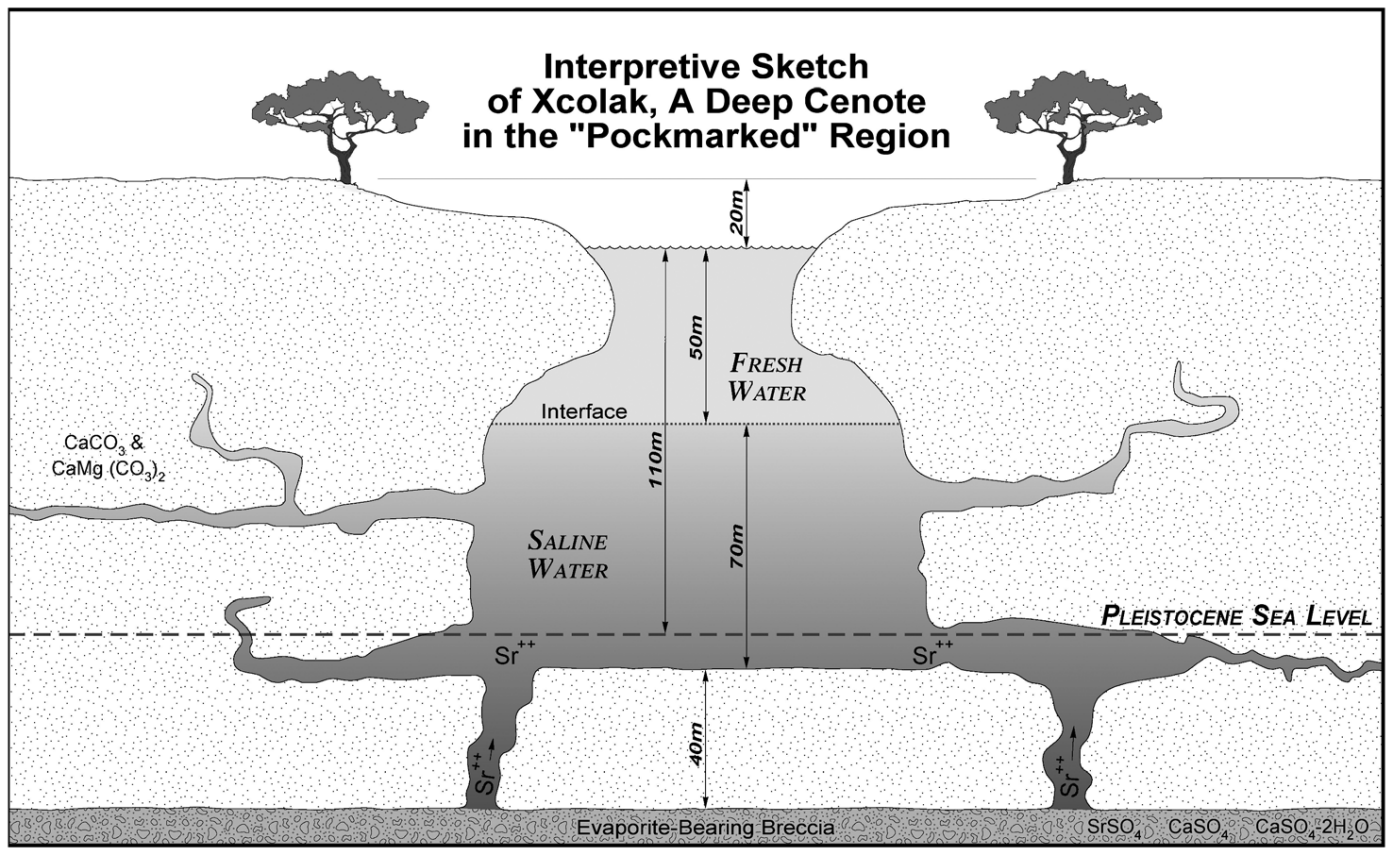
Interpretive sketch showing geological setting and variation in salinity with depth. Illustration courtesy of Gene Perry and Mark Howland.

The sample chambers were placed in Sinkhole (locally known as a Cenote) Xcolak, located in the northern Yucatan Peninsula [Figure 1], for 3 to 4 months. Deep cenotes (sinkholes) are common in the northern Yucatan due to the Late Cretaceous Chicxulub impact crater [18], where the impact fractured the underlying carbonate bedrock and allowed for increased hydrogeologic processes and resulting karst structures, such as caves and cenotes. The cenote used in this study was chosen based on its availability and well-documented geochemical and hydrogeologic characteristics [18], [19]. Cenote Xcolak is approximately 75 m in diameter and 120 m deep [Figure 2], and stratified. The upper boundary of the saltwater interface is located approximately 52 m below the water surface, as indicated by the increase in electrical conductivity. Dissolved oxygen content decreases below 0.5% at the upper zone of the saltwater interface, indicating an oxic to anoxic condition transition due to sulfate reduction [19]. The stratification of Cenote Xcolak allowed experiments on microbial interaction to be conducted for bones in a variety of environmental conditions.

Bone samples from *G. gallus* were placed in Cenote Xcolak from December, 2008 to March, 2009, and *A. mississippiensis* samples were placed from July, 2009 to November, 2009. After retrieval, bones were vaccuum sealed and stored on ice until returned to Northern Illinois University where they were stored at −70°C until analysis. Bones of *G. gallus* were lypholized and cut into four pieces and prepared for phospholipid fatty acid analysis (PLFA), Scanning Electron Microscopy-Energy Dispersive Spectroscopy (SEM-EDS) analysis, ethylenediamenetetraacetic acid (EDTA) demineralization, and light microscopy. Sectioning was performed using a laminar flow hood, a set of sterilized forceps, and 20 flame-sterilized cutting blades. A new flame-sterilized blade was used for each sample and the forceps were re-sterilized prior to cutting each specimen. All specimens were placed in labeled sterile plastic bags and placed in a −70°C freezer until further analysis.

Phospholipid fatty acid (PLFA) extraction and analyses were performed on the *G. gallus* bones retrieved from the cenote to identify variations in active microbial communities in the bones from different intervals of the cenote and under different preservational conditions [20] (slightly modified from the 2006 PLFA training outline organized by Zhanna Yermakov from Argonne National Laboratory, Argonne, IL).

Small fragments of dinosaur bone were obtained from three groups of Hell Creek dinosaurs. These specimens include bone samples from a theropod (BRM P2002.4.1), a ceratopsian (BMR P2006.4.1), and a thescalosaur (BMR P2006.4.309). Exact coordinates of the collection sites for the specimens are on file in the paleontology collections at the Burpee Museum of Natural History, Rockford, Illinois (BMR), where the specimens are housed. Samples were chosen based on two categories; 1) a high volume of trabecular bone in the sample, and 2) specimens that had not been directly treated with polyvinal acetate (“vinac”), a common field preservant. Tissues were demineralized in plastic containers with 30–50ml of 0.5M EDTA (pH 8.0) for two weeks, changing the acid every three days. EDTA was prepared by dissolving 186.1g of EDTA crystals in 800mL of de-ionized water. The pH was adjusted to 8.0 by adding approximately 20g of NaOH pellets, and brought to 1L volume with de-ionized water. The solution was then autoclaved and stored at room temperature. The pH was measured using a Corning pH/Ion analyzer 455.

An initial test was performed to de-mineralize dinosaur bone, however an additional step was added to avoid draining out extracts; samples were centrifuged at 2500 RPM for 3 minutes prior to changing the acid. This process physically broke down the delicate structures extracted from the bones. In the later experiments, this step was omitted.

After EDTA demineralization, the specimens were rinsed with a balanced phosphate buffer or Ringer solution (recipe as described in collagenase from *Clostridium histolyticum* C2799, Sigma-Aldrich) and imaged using a Heerbrugg WILD M3 Stereomicroscope at 6X- 16X- 40X magnifications with a digital camera. Samples were also prepared for SEM and EDS analysis. Samples were allowed to air-dry and then mounted to aluminum stubs by either SEM Sticky Tabs (Canemco) or polyvinyl acetate glue. Specimens were then gold-coated to a thickness of 300–400 Ångstroms, using a Polaron E5150 Film Thickness Controlled Sputter Coater. Specimens were then imaged in a JEOL JSM5610LV Environmental SEM. Elemental scans were obtained with a Thermo-Noran Vantage DSI X-ray microanalyzer coupled to the SEM.

Because museum specimens may have had some contact with fossil preservatives such as polyvinyl acetate and cyanoacrylate, though not apparent in the sample, a control sample was prepared consisting of a small fragment of dinosaur bone that was intentionally coated in polyvinyl acetate and then demineralized in EDTA and imaged. Polyvinyl acetate was also prepared on an SEM stub for SEM and EDS analysis.

### Statistical Analysis

To investigate variation of microbial community structures in bones exposed to different environments and under different taphonomic conditions, a Principle Component Analysis (PCA) was performed using PC-ORD (MjM Software Design, Gleneden Beach, OR). PCA is a statistical technique for identifying patterns in data, and expressing data in a way that highlights their similarities and differences [21].

**Figure 3:**
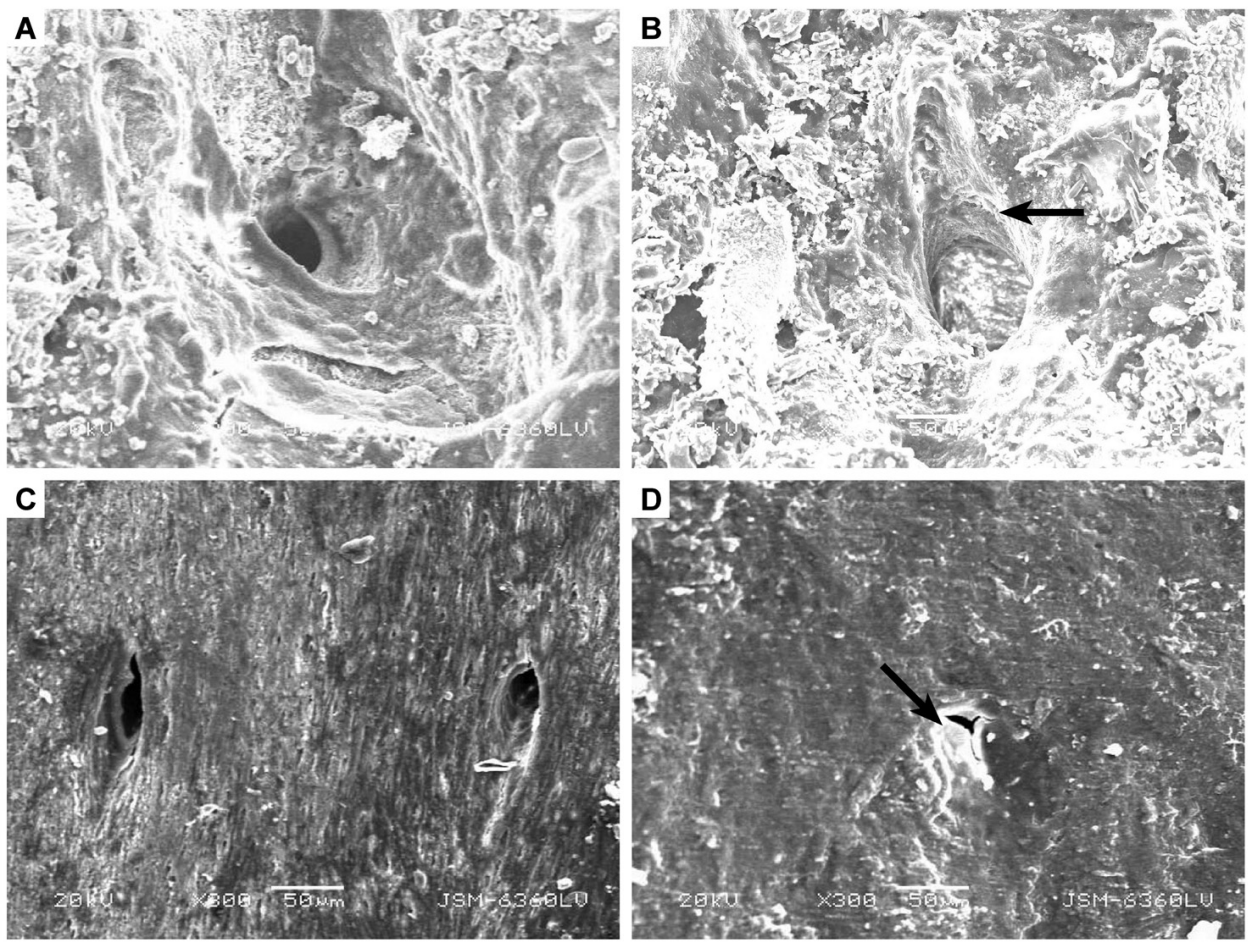
Examples of biofilm infiltration classes for extant archosaur bone. A) Class 0: a pore on a fractured bone sample from *A. mississippiensis* showing no biofilm infilling; B) Class 1: a pore on a fractured bone sample from *A. mississippiensis* showing a biofilm coating the inner pore wall; C) Class 2: pores from a whole bone sample of *A. mississippiensis* showing a significant biofilm coating on the inner pore walls; D) Class 4: a pore from a fractured bone sample of *G. gallus* showing a complete infiltration or covering of the pore. All images shown at 300× magnification.

In order to quantify the degree of modern biofilm infiltration in extant archosaur bone, pores were identified on the surface of SEM samples at a magnification of 300×. Pores were categorized by size (µm) (Table 1) and degree of biofilm infiltration (Table 2, Figure 3). Kolmogorov-Smirnov Goodness-of-fit tests [22] were performed on the pore size/infiltration matrix to identify variation in pore sizes among extant archosaur samples, and to observe variation in biofilm infiltration among samples in different taphonomic conditions at a 0.05 significance level.

**Table 1:** Categorization of observed pore sizes in extant archosaur bones and associated biofilm growth.

Classification	Pore Size (µm)
1	0–25
2	26–50
3	51–75
4	76–100
5	101–125
6	126–150
7	151–175
8	176–200
9	201–225
10	226-up

**Table 2:** Degree of biofilm infiltration into observed pores in extant archosaur bones.

Pore Infiltration	
0	No biofilm infilling pore
1	Biofilm coating inner pore walls
2	Thick biofilm coating or infilling
3	Complete/Near complete biofilm infilling

## Results

### The Characteristics of Biofilms and Microbial Communities in Extant Archosaur Bones

**Figure 4:**
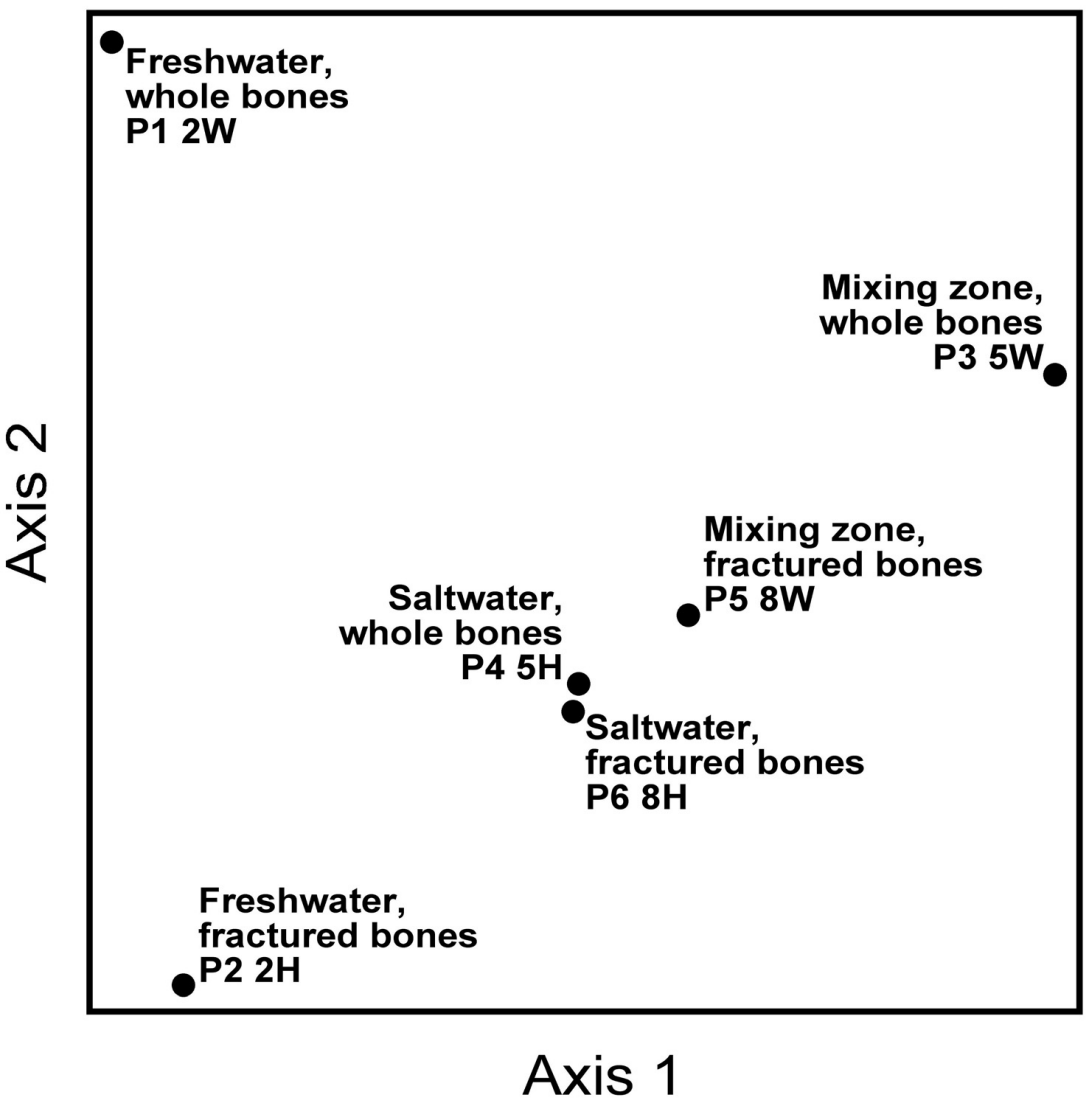
Results of PC-ORD statistical analysis. Testing for significant differences in the amount of PLFA assemblages in each sample, these results show a significant difference between samples of whole bone from aerobic conditions (2W) and fractured bone from aerobic conditions (2H), suggesting a difference in microbial communities due to bone condition prior to placement in the cenote. Axis 1 is based on variation in cenote environmental conditions, while Axis 2 is based on whole or fractured bones.

**Figure 5:**
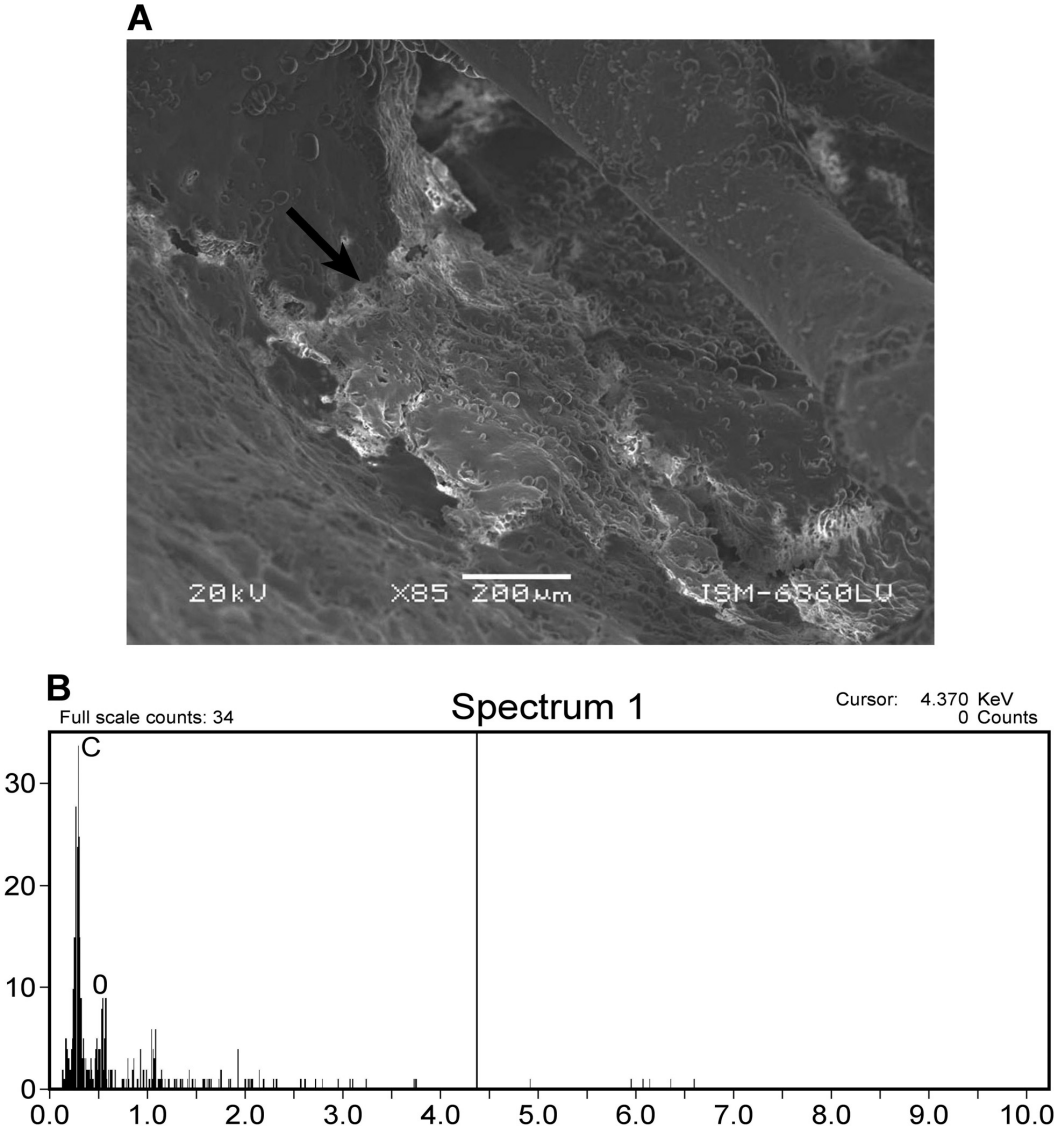
SEM and EDS results of *G. gallus* control sample. A) SEM image of primary soft tissue in *G. gallus* control sample with primary blood cells observable. B) EDS results of primary soft tissue in *G gallus* control sample showing a high abundance of carbon.

Bone samples were analyzed to identify the degree of biofilm growth and infiltration, and to investigate variation in microbial communities with different preservational settings. To investigate the degree of biofilm growth on bones, samples were sectioned for SEM analysis and surface pores were counted, measured, and assessed for biofilm infiltration. Kolmogorov-Smirnov Goodness-of-Fit tests show no significant difference in the distribution of pore sizes among archosaurs (Figure 4A). However, significant differences were found in the degree of microbial infilling of extant archosaur bone pores related to taphonomic conditions (Figure 4B). Variation in microbial community was assessed by PLFA analysis and Principle Component Analysis (PCA) and show significant differences in microbial communities between whole and bisected *G. gallus* bones in freshwater environments (Table 3, Figure 5).

**Table 3:** Bone samples and PLFA results.

	PLFA										
Samples	i16:0cis9	4:0	17:0	18:0	21:0	23:0	10:0	11:0	14:cis9	14:0	i16:0
**Freshwater_whole_**	0.4839	ND	ND	0.45699	ND	ND	ND	ND	ND	0.591	ND
**Freshwater_fractured_**	0.0001	0.0379	5.41E-05	0.943	0.0021	5.53E-05	0.0012	0.0016	ND	ND	0.0157
**Mixing zone_whole_**	ND	0.9623	ND	0.0376	ND	ND	ND	ND	ND	ND	ND
**Mixing zone_fractured_**	ND	0.4447	ND	0.4992	ND	ND	ND	0.0517	0.0042	ND	ND
**Saltwater_whole_**	ND	0.5755	ND	0.4244	ND	ND	ND	ND	ND	ND	ND
**Saltwater_fractured_**	ND	0.4454	ND	0.5292	ND	ND	ND	0.0029	ND	ND	0.0224

Biofilm growth on bones varies with taphonomic conditions. No discernable differences in biofilm growth were noted between whole-bone specimens of either *A. mississippiensis* or *G. gallus*, nor were any discernable differences identified between halved-bone specimens from the two respective taxa. However, as clarified by statistical analyses, the degree of biofilm infiltration into surface pores was significantly higher in whole-bone samples than bisected samples in both taxa.

**Figure 6:**
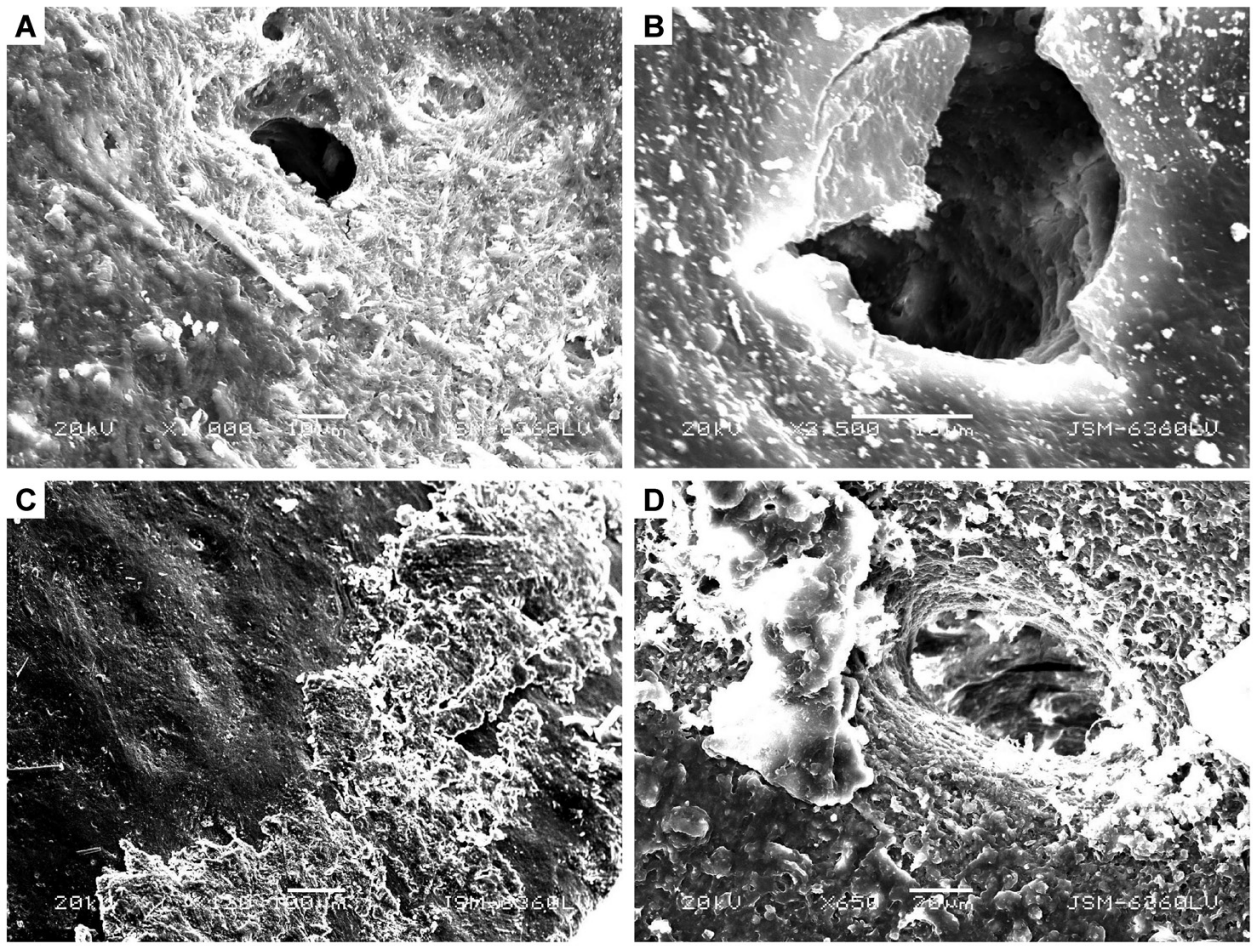
Biofilm growth on an extant *G. gallus* bone. A) Biofilm surrounding surface pore. B) Biofilm partially covering a surface pore and lining the inner pore surface. C) Encrusting biofilm on the surface of a bone. D) Biofilm on the surface of a bone and on the inner pore surface.

**Figure 7:**
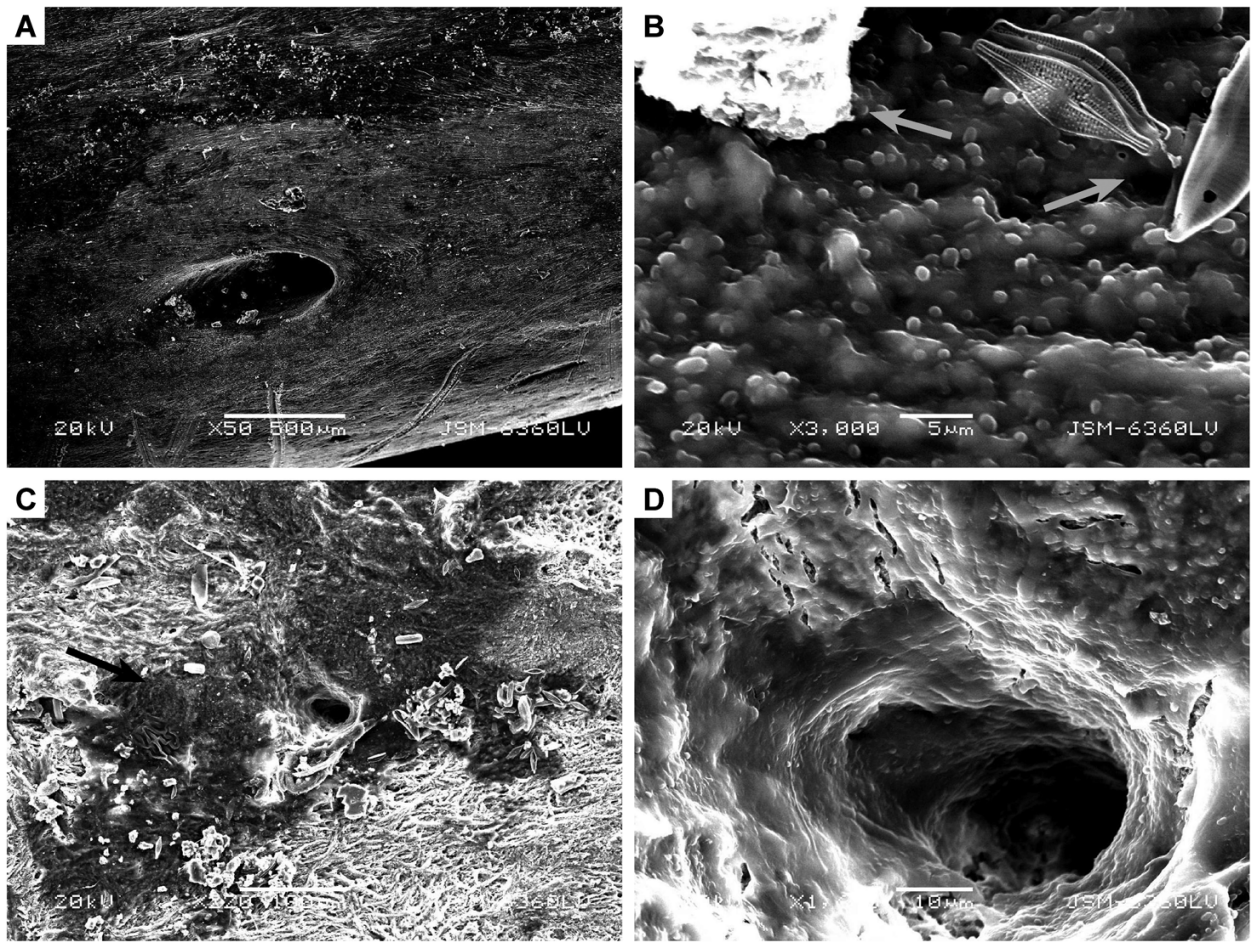
Higher magnification SEM images of biofilm growth on extant *G. gallus* bone. A) Biofilm on inner pore surface. B) Higher magnification of Figure 7A, showing individual bacterial cells and EPS. Debris and diatoms are adhered to the biofilm. C) Biofilm surrounding surface pore and clean bone surface. D) Higher magnification of Figure 7C, showing biofilm on inner pore surface.

**Figure 8:**
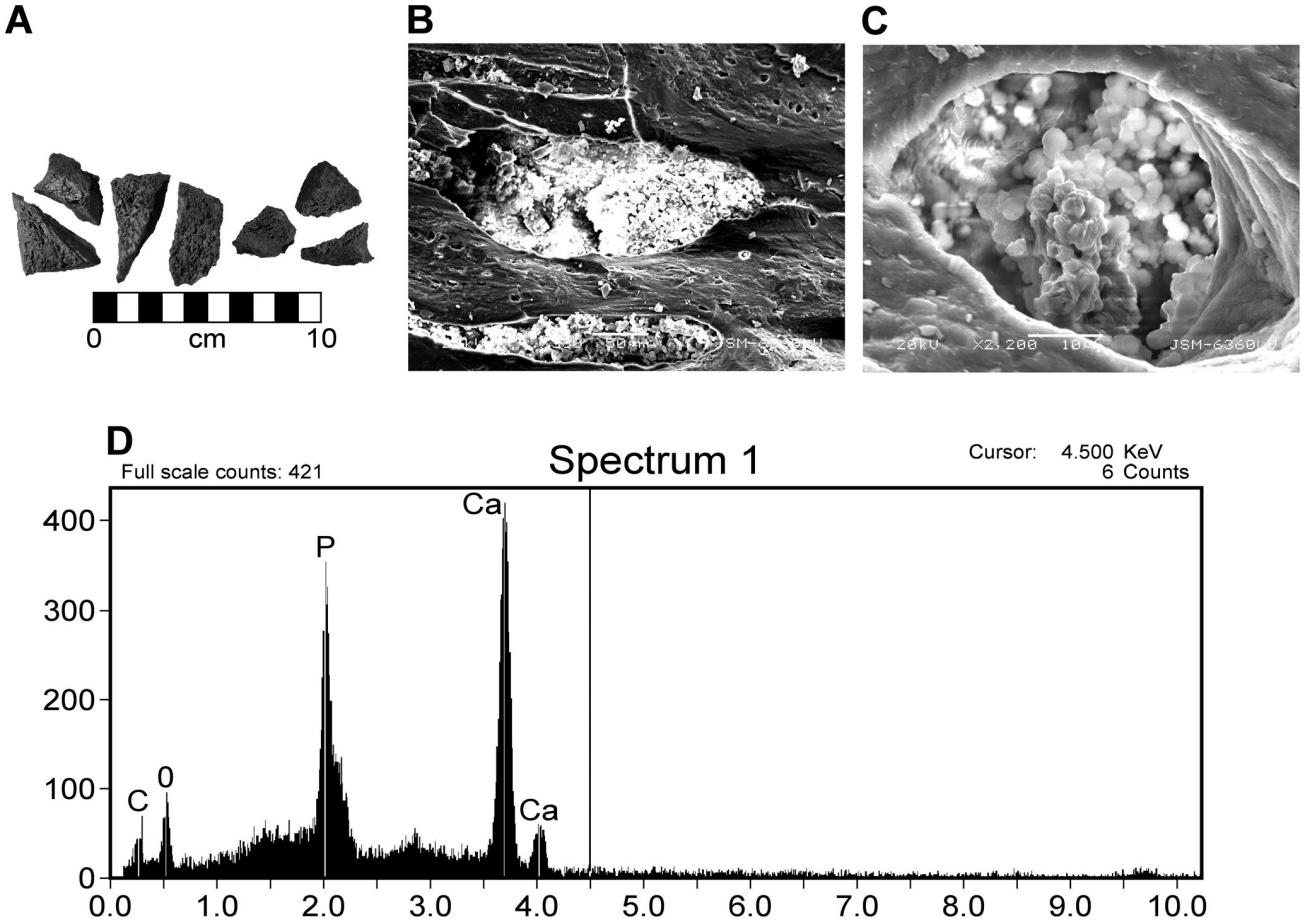
Results of thescelosaur specimen BMR P2006.4.309. A) SEM images of thescelosaur samples prior to demineralization. Scale bar equals 10 cm. B) Thescelosaur samples at 330× magnificaiton, showing bacterial cells filling the surface pores. C) Thescelosaur samples at 2200× magnification, showing the mineralized bacterial cells in fossil specimens. D) EDS results of the mineralized bacterial cells in fossilized thescelosaur samples, showing a Ca- and P-rich signature.

Since both primary soft tissue and microbial biofilms are composed of organic carbon, control samples and biofilm samples possess a similar EDS signature that is unreliable for differentiation. However, primary soft-tissues in control samples are morphologically different from biofilm samples. Primary soft-tissues analyzed by SEM show red blood cells and vessels at relatively low magnifications (<100×) (Figure 6) whereas biofilms on bones shows smooth, undulating surfaces at similar magnifications (Figures 7, 8). Bacterial cells and EPS are clearly visible at much higher magnifications.

### Structures Present in Fossil Dinosaur Bones Before and After Demineralization

**Figure 9:**
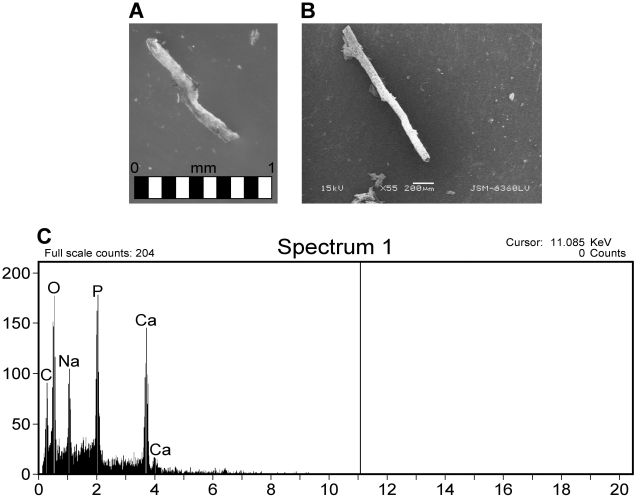
Thescelosaur bone sample extracts after complete demineralization in EDTA. A) Light microscopy image of branching, fragile, semi-transparent vessels remaining after demineralization. Scale bar equals 1 mm. B) SEM image of thescelosaur extract, at 55× magnification. C) EDS analysis of frangible thescelosaur extracts, rich in calcium an d phosphate.

**Figure 10:**
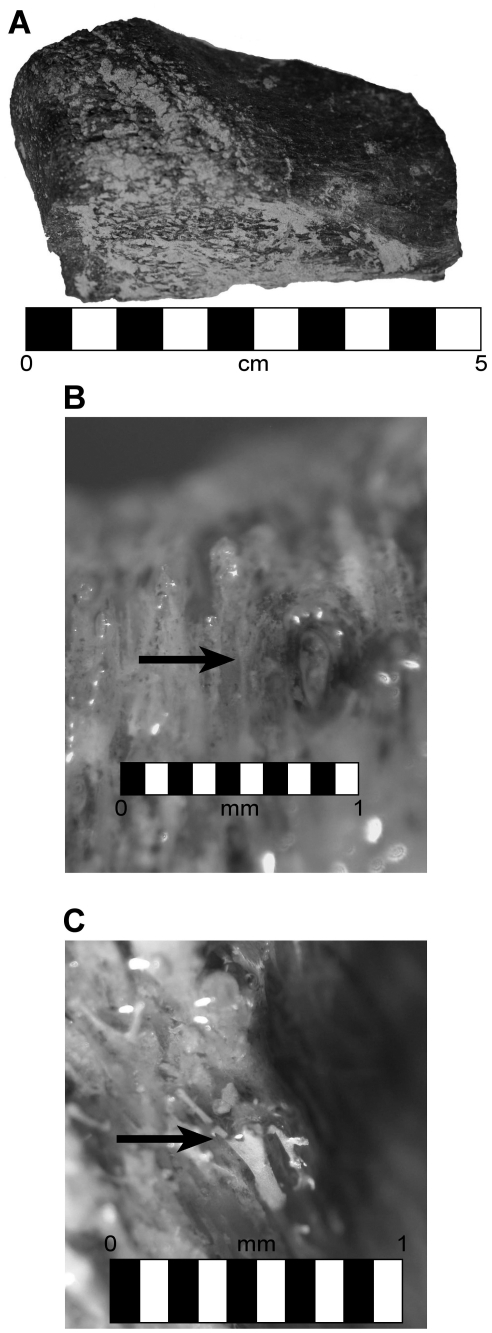
Results of theropod specimen BMR P2002.4.1. A) Theropod bone sample prior to EDTA demineralization. Scale bar equals 5 cm. B) Partial demineralization of theropod sample, showing “etched” vessels released during demineralization. Scale bar equals 1 mm. C) Release of resistant vessels from fossil bone. Scale bar equals 1 mm.

Samples of Late Cretaceous thescelosaur bone were sectioned and analyzed in the SEM prior to EDTA demineralization (Figure 9). The surface pores of the fragments were filled with small spheres approximately 1µm in diameter, consistent in shape and size to bacterial cells. EDS analysis of these spheres illustrate and Ca- and P-rich signature, and upon higher magnification, reveal mineralized spheres within the pores. After complete demineralization of thescelosaur samples, small semi-transparent Ca- and P-rich vessels were recovered from the acid baths (Figure 10). These vessels appear morphologically similar to extracts described by previous authors [1], [4].

**Figure 11:**
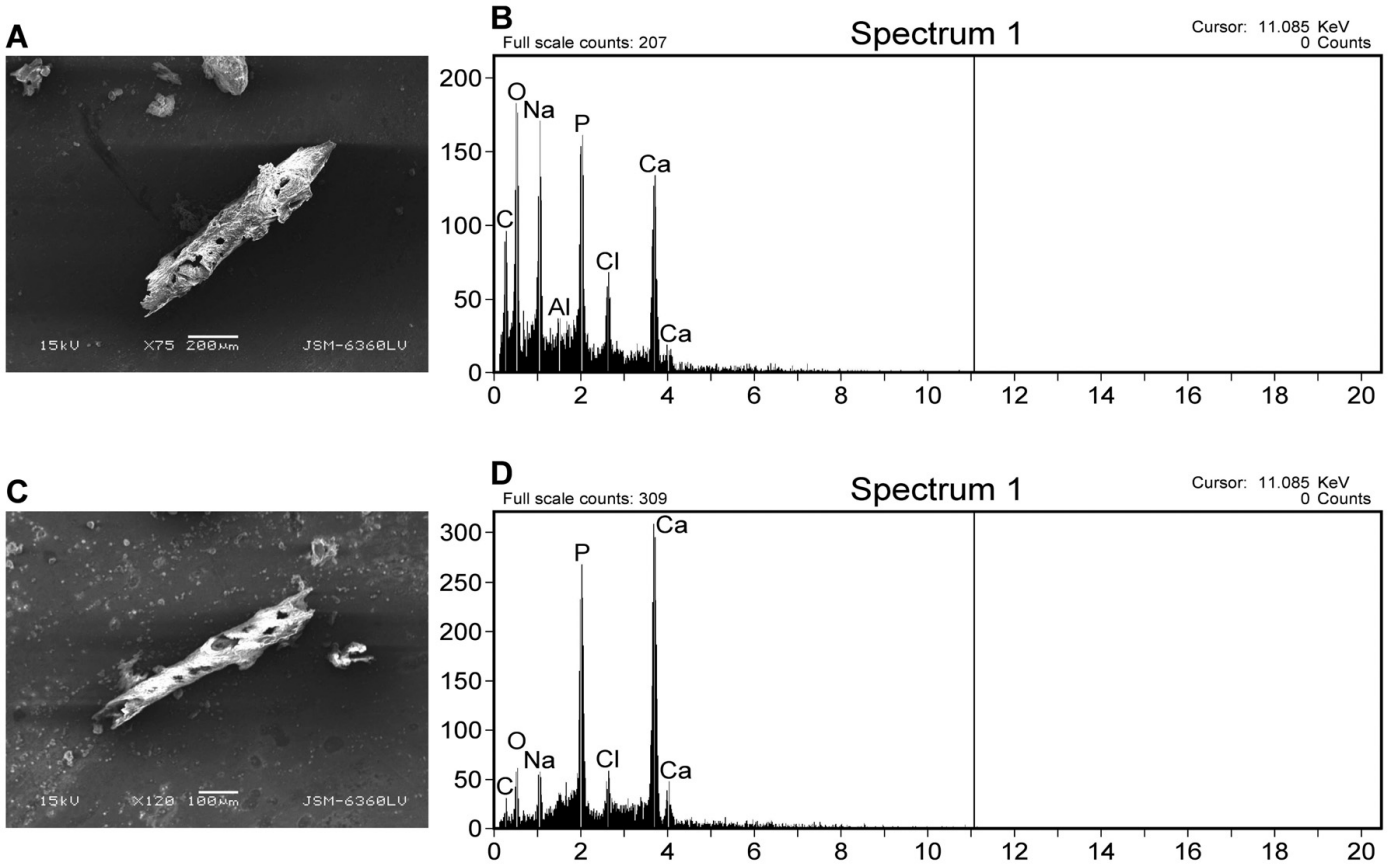
Complete demineralization of theropod bone. (A) SEM image of hollow, vascular theropod extract at 75× magnification. B) EDS signature of 11(A) vessel, rich in Ca, O, Mn, and P. C) SEM image of theropod vessel at 120× magnification. D) EDS signature of 11(C) vessel, rich in calcium and phosphate.

**Figure 12:**
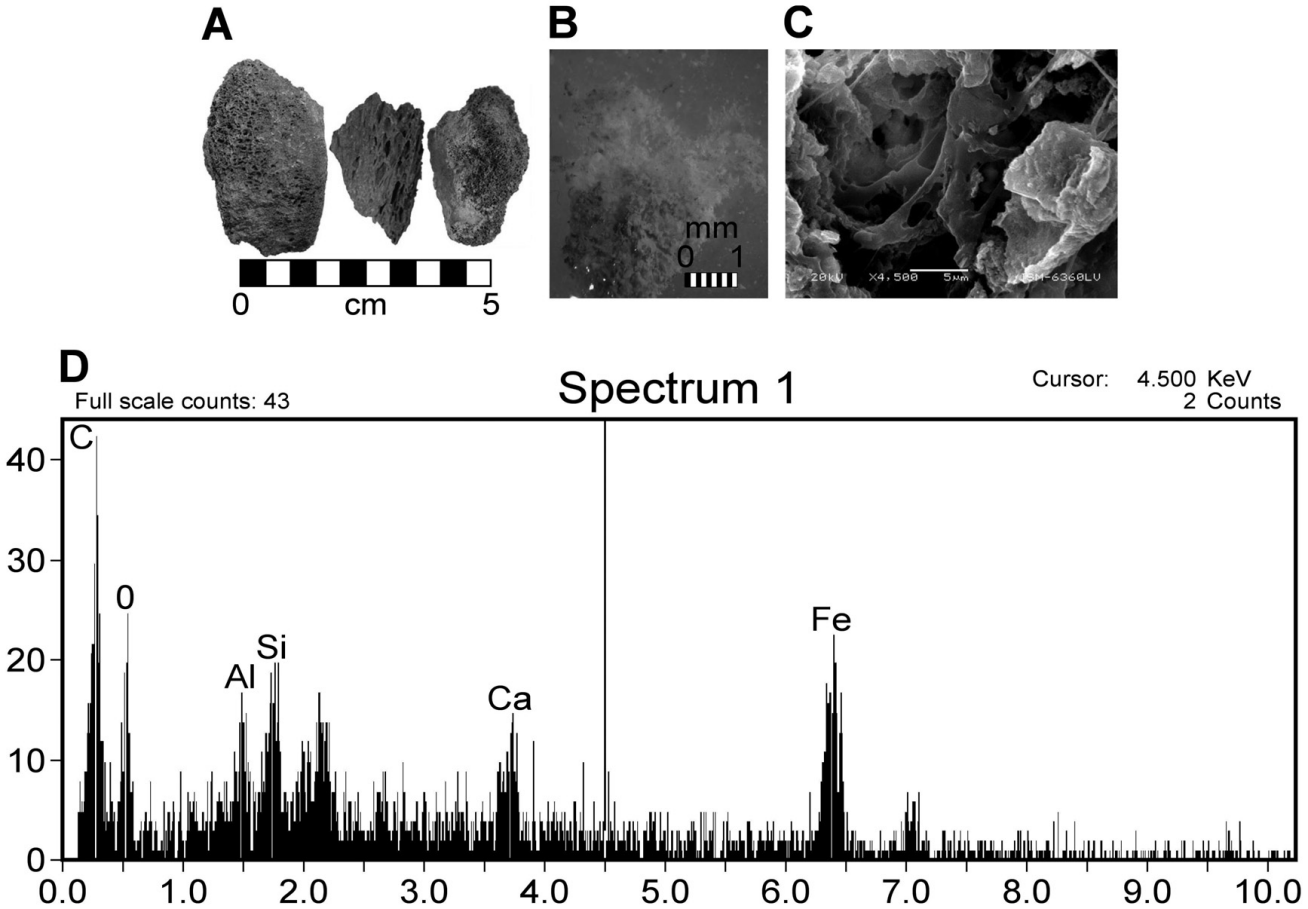
Ceratopsian specimen BMR P2006.4.1 bone fragments. A) Bone fragments prior to demineralization. Scale bar equals 5 cm. B) Light microscopy image of ceratopsian sample after demineralization, revealing a network of pliable, how vessels and tubes. Scale bar equals 1 mm. C) SEM image of pliable ceratopsian extracts at 4,500× magnification. D) EDS signature of SEM image 12(C), high in carbon, iron, and silica.

Fragments of theropod bone were demineralized to completion as well. Prior to complete demineralization, fragments were removed from the acid baths and imaged to illustrate the “etching out” of resistant branching, vessel-like structures from the mineral component of bone (Figure 11). These structures are morphologically consistent with previous studies [1], [4]. After complete demineralization of theropod bone (Figure 12), the remaining branching, vessel-like structures were analyzed in the SEM and showed a Ca- and P-rich EDS signature.

**Figure 13:**
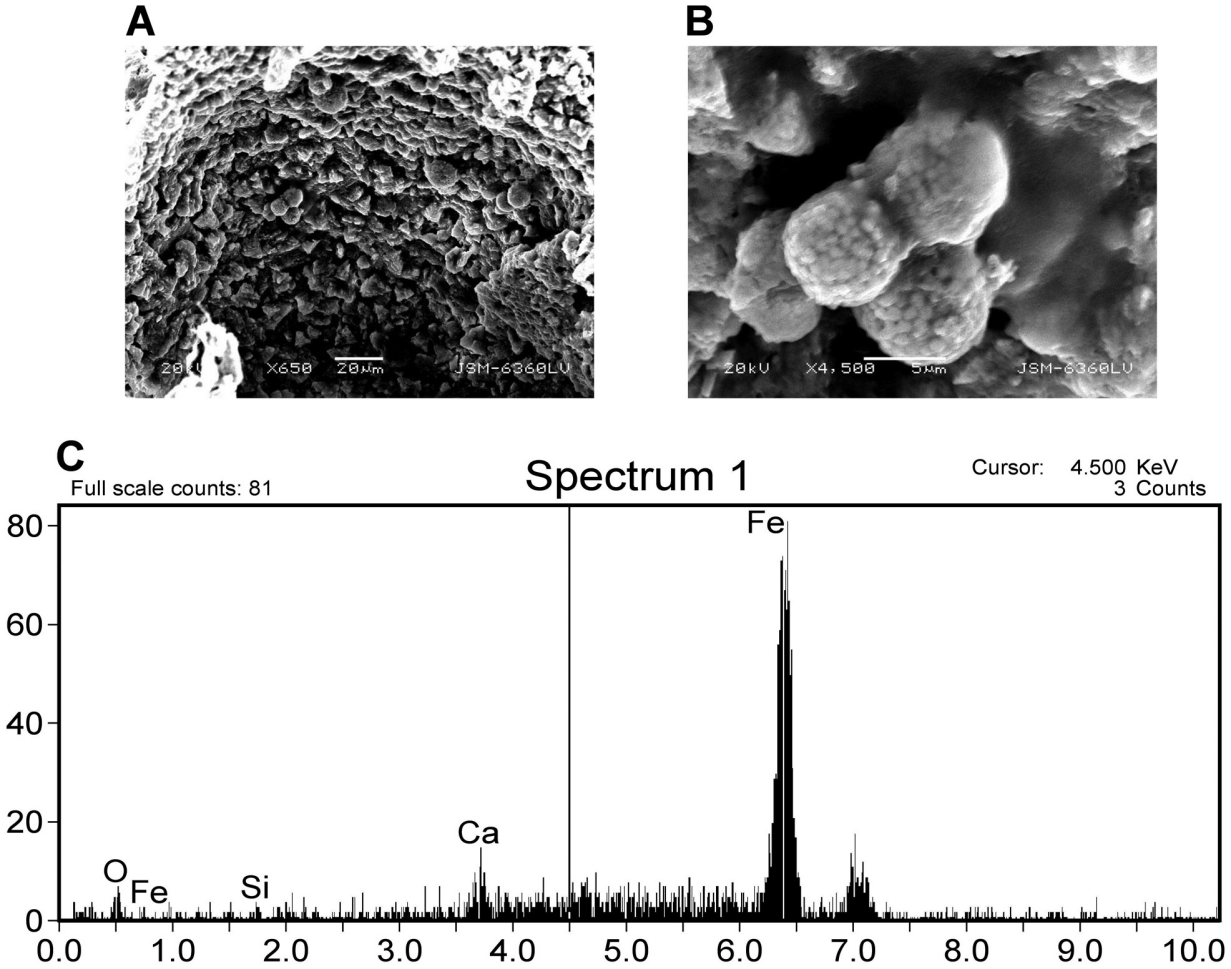
SEM and EDS results of ceratopsian specimen BMR P2006.4.1. A) SEM image at 650× magnification of pliable ceratopsian vessels. B) SEM image at 4,500× magnification of framboids identified in pliable ceratopsian vessels. C) EDS signature of framboids, rich in iron.

**Figure 14:**
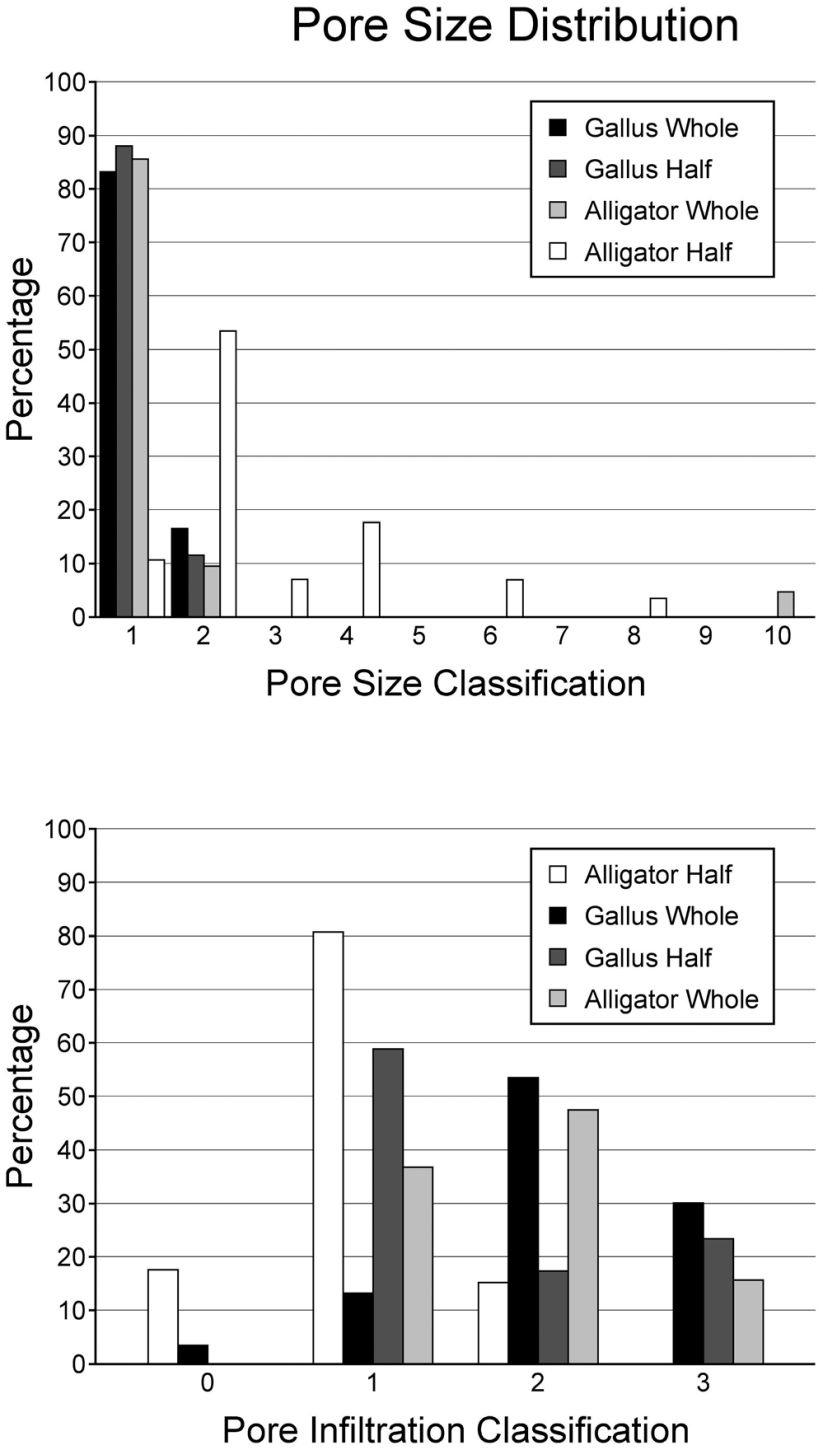
Histograms of (A) pore size distributions and (B) pore infiltration distribution among extant archosaur samples.

The ceratopsian bone fragments (Figure 13) yielded strikingly different structures from hadrosaur and theropod samples. After complete demineralization, the ceratopsian remains consisted of transparent, pliable, vessel-like structures similar to those described by previous authors [1], [4]. These structures showed an EDS signature that was high in carbon, iron, possessed some silica. Furthermore, the ceratopsian extracts also possessed small, spherical structures with a high iron EDS signature (Figure 14). Similar structures were previously noted in a demineralized turtle carapace from the Hell Creek Formation and identified as pyritic framboids [4].

## Discussion

The influence of bacteria on extraordinary preservation (i.e. lagerstatten) has been discussed [23]–[27]. However, these findings are at odds with traditional inferences of microbes as destructive agents due to biodegradation [10]. Previous reports have illustrated the crucial role of microorganisms in the early stages of fossilization through rapid mineralization of soft-tissues [28]. The ability of microbial biofilms to retard decomposition of delicate soft-tissues by means of mineralization has been documented [29]–[31], though previous studies have dealt primarily with microbially-mineralized soft-tissues, such as phosphatization of soft-tissues in the fossil record, focusing on the microbial transformation of soft-tissues into authigenic minerals [12].

The preservation of embryonic structures due to early biofilm mineralization has previously been reported [30]. Although the soft-tissue nature of embryos does not normally fossilize well, the mineralization of biofilms in the earliest biostratinomic stages can yield high-detailed textures of the original soft-tissues.

Phosphatized soft-tissues in the basal ornithimimosaur, *Pelecanimimus polydon*, have also been described in the form of muscle and skin [25]. The results of this study strongly suggest that microorganisms play a role in the preservation of primary soft-tissues in vertebrate organisms, without extensive secondary mineralization.

Differences in morphological and elemental characteristics in fossil extracts evaluated here suggest that microbial infiltration into bones may heavily impact internal preservation in fossil archosaur bone. Statistically significant differences are present between microbial communities and degree of biofilm infilling of surficial pores based on whole versus halved bones regardless of taxa, supporting the hypothesis that preservation is strongly influenced by early biostratinomic processes. In this case, halved bones are easier for microorganisms to infiltrate and metabolize soft-tissues; the system is much more open. However, bacteria can only infiltrate whole bones via natural openings (pores or foraminae) [15]. These cavities act as microenvironments in which the biofilm establishes, metabolizes available primary tissues, and mineralizes, as evident from EDS analyses of fossil extracts. This mineralization walls off access to more internal organic material, limiting further penetration and metabolization of the interior of the bone in a process we refer to as “microbial masonry”. Thus, fractured bones have a less-likely chance of preserving primary soft-tissues because microbial masonry can only occur where access to organic matter is available exclusively via microscopic forminae.

Recognizing evidence of the microbial process has implications for reconstructing taphonomic histories. For example, the thescelosaur samples analyzed possessed mineralized biofilms in pore spaces before and after demineralization. However, the bone was discovered fractured *in situ*, suggesting that fracturing was the result of biostratinomic processes or diagenetic deformation. Fossil vertebrates that possess a large amount of frangible extracts after demineralization could indicate a complex taphonomic history in which the bone was primarily unfractured when microorganisms began establishing biofilms on the surface and infiltrating surface pores, followed by mineralization and bone fracturing due to biostratinomic processes such as trampling [32] or diagenetic processes, such as compaction due to burial.

The thescelosaur and theropod specimens examined were both obtained from fractured *in situ* bones discovered in fine sandstone facies of the Hell Creek Formation in southeastern Montana, USA. The ceratopsian fragments were from an unfractured bone obtained from a mudstone facies of the same region. The degree of compaction of sediments during lithification could control the rate of post burial diagenetic fracturing and crushing of fossil bone. Mudstone lithification involves more compaction than sandstone lithification, altering fossil bones respectively. As such, one would expect decreased crushing in bones obtained from sandstone facies and thus a higher degree of soft-tissue preservation despite having higher groundwater throughflow. This result is at odds with the raw data from previous studies on *T. rex* and *B. canadensis* specimens [1], [3].

However, in the experiments reported here, the extracted pliable tissues were obtained from a sample encased in mudstone. Although many of the ceratopsian bones were fractured syndepositionally, the high clay content and decreased permeability of the matrix may have acted as an extra barrier, decreasing available oxygen, groundwater flow, and ultimately retarding microbial action.

Although microorganisms are traditionally thought to rapidly metabolize and destroy organic material, decreasing the chances for fossilization, this study has shown that not only may microorganisms play an integral role in the formation of fossils by mineralizing soft tissues, but also that in some cases biofilms may directly enhance the preservation of vertebrate primary soft-tissues.

The results of this study indicate that exquisite preservation of pliable soft-tissues may be related to a microbial masonry process whereby the formation of microbial biofilms wall off internal surfaces of bones during early taphonomic stages. These biofilms metabolize organic materials and mineralize, forming resistant structures or microbial masonry wall surfaces across internal pores openings in bones. These results have potential to allow for more detailed taphonomic reconstructions and contribute to a more nuanced understanding of fossil preservation in the form of soft-tissues structures and biomolecules.

The claim of the presence of primary soft-tissues in fossil vertebrates has been supported by the identification by mass spectroscopy of biomolecules in the form of collagen and proteins [2], [3]. However, these studies have failed to produce a potential mechanism for the preservation of soft tissues throughout geological time. The results presented here suggest an important role of microorganisms in taphonomic processes, notably for the preservation of primary soft-tissues within bone, through a microbial masonry process. Although biomolecular studies on soft-tissue extracts from fossil vertebrates have shown that the proposed alternative interpretation of primary soft-tissue as microbial biofilms is unlikely [2], [3], biofilms may play a critical role in the preservation process.
